# Acknowledgment to Reviewers of *Metabolites* in 2021

**DOI:** 10.3390/metabo12020182

**Published:** 2022-02-15

**Authors:** Metabolites Editorial Office

**Affiliations:** MDPI AG, St. Alban-Anlage 66, 4052 Basel, Switzerland

Rigorous peer-reviews are the basis of high-quality academic publishing. Thanks to the great efforts of our reviewers, *Metabolites* was able to maintain its standards for the high quality of its published papers. Thanks to the contribution of our reviewers, in 2021, the median time to first decision was 13 days and the median time to publication was 38 days. The editors would like to extend their gratitude and recognition to the following reviewers for their precious time and dedication, regardless of whether the papers they reviewed were finally published:
Abbara, AliLázaro, IolandaAbdallah, Mohamed F.Le Barz, MélanieAbdel-Aziz, H. M. M.Lee, ByungjuAbdelhamid, Hani NasserLee, Chien-HungAcharjee, AnimeshLee, Do YupAdamski, JerzyLee, Eun YeolAdav, Sunil S.Lee, NaraeAdewole, DeborahLee, Ting-IAdighibe, OmanmaLee, Wen-ChinAdnan, MohdLee-Sarwar, Kathleen AnneAfonso, ClaudioLefevre, SjannieAgliassa, ChiaraLeglize, PierreAlAkwaa, FadhlLegrand, François-XavierAlam, MaroofLemaitre, Rozenn N.Alarcon, JulioLengyel, ImreAlbreht, AlenLetek, MichalAl-Dujaili, EmadLi, Chia-JungAlessio, NicolaLi, GuohuiAlexandrova, ElenaLi, SunanAlexovič, MichalLi, XiangnanAlexy, TamasLiao, Hsiao-WeiAllen, ScottLiao, Jyh-feiAllende, Maria LauraLiao, PanAlloza, IraideLiapi, CharisAlonso, Rosa MariaLichtenberg-Kraag, BirgitAl-Robaiy, SamiyaLighezan, DanielÁlvarez-Sola, GloriaLigor, MagdalenaAlvarez-Suarez, JoseLim, Hyun GyuAlves, ElianaLin, FanAlygizakis, NikiforosLindon, John C.Amarowicz, RyszardLindquist, Diana M.Amat, ConcepcióLinial, MichalAmer, BasharLipinski, PawelAmir, RachelLipiński, Piotr F. J.Amoutzias, GrigorisLipkova, JanaAndersen, Maria KarolineLitvinova, LarisaAndreani, GiuliaLiu, Chung-JungAnesi, AndreaLiu, LiezhaoAngelidi, AngelikiLiu, ZhaominAngelini, CorradoLlabrés-Segura, MercèAngom, Ramcharan SinghLocci, EmanuelaAnnunziata, Maria GraziaLoiseau, NicolasAns, MuhammadLojkova, LeaAntonkiewicz, JacekLombardi, PieroApaza Ticona, LuisLondero, Ambrogio P.Apidianakis, YiorgosLong, Nguyen PhuocAragonés, JuliánLoniewski, IgorArapitsas, PanagiotisLopaschuk, Gary D.Araújo, Ana MargraidaLopes, DianaArcher, StephanieLópez Malo, DanielArdalani, HamidrezaLópez-Medrano, RamiroArias Del Castillo, NataliaLopitz-Otsoa, FernandoArita, MasanoriLorenzo, Jose M.Ariyawansa, HiranLorenzo, Petra I.Armesto-Alonso, SusanaLorkiewicz, Pawel K.Aron, Allegra T.Łotocka, BarbaraArtati, AnnaLovre, DraganaArtunc, FerruhLu, Chi-YuArtyukhin, AlexLu, LingengArunkumar, AbhiramLucchetti, JacopoArús, CarlesLuchinat, ClaudioAsano, NaokiLuddi, AliceAsnicar, FrancescoLukowski, JessicaAssy, NimerLusczek, Elizabeth R.Astl, JaromírLutter, DominikAtlante, AnnaLux, AnjaAttardo, Geoffrey M.Ma, HongwuAuchtung, JenniferMa, SihuiAujayeb, AvinashMaekawa, MasamitsuAvesani, LindaMaffia, MicheleAzad, Obyedul KalamMaicas, SergiBąchor, RemigiuszMaioli, MargheritaBadiani, MaurizioMajera, DusanaBaenas, NievesMajumder, Erica L.Baidoo, EdwardMalcangi, GiuseppinaBairaktari, Eleni T.Malmendal, AndersBaj, JacekMaloberti, AlessandroBajguz, AndrzejMalusa, EligioBaldanzi, GianlucaManai, FedericoBalgoma, DavidMandatori, DomitillaBalogh, GáborManganas, PhaneeBaloni, PriyankaMangiaterra, GianmarcoBanerjee, DeepanwitaManickam, RavikumarBanerjee, KalpitaManier, Sascha K.Bankova, VassyaMannell, HannaBao, YongmingMansueto, GelsominaBaragetti, AndreaMäntylä, ElinaBarberis, ElettraMaráková, KatarínaBarbier-Torres, LucíaMarco-Rius, IreneBargiela, AriadnaMare, RosarioBarrott, JaredMarginǎ, DenisaBarshtein, GregoryMarín De Mas, IgorBartnik, MagdalenaMarini, Herbert RyanBarton, Gregory P.Markel, ArcadyBasit, AbdulMarkova, SilviaBasler, GeorgMartín Espinosa, Noelia MaríaBasu, ReetobrataMartin, Hector GarciaBasu, SaumikMartin, Luc J.Beale, DavidMartín-Cuervo, MaríaBeck, SarahMartinez-Hervas, SergioBeis, DimitrisMartínez-Nicolas, Juan JoséBel′skaya, LyudmilaMartino, AlessioBeloborodova, Natalia V.Martin-Sabroso, CristinaBelter, AgnieszkaMasino, FrancescaBen-Amotz, AmiMaskell, PeterBéné, Marie C.Mason, ShaunBenina, MariaMaspero, CinziaBentel, Jacqueline M.Massiot, GeorgesBerezin, AlexanderMastana, SarabjitBernillon, StéphaneMataragas, MariosBernstein, NiritMathiyalagan, PrabhuBertram, Hanne ChristineMatsubara, TakumaBetekhtin, AlexanderMatsuda, FumioBetti, MarcoMattonen, SarahBeuriat, Pierre-AurélienMatuszewska, AgnieszkaBeversdorf, DavidMaudens, KristofBezuglov, EduardMaurea, NicolaBhattacharya, BhaswatiMaurer, FelixBhawal, RuchikaMauricio, Maria DoloresBiagi, MarcoMauro, AnnunziataBiagini, GiuseppeMayrhofer, Patrick M.Bianco, CarmelinaMazilu, LauraBianco, FrancescoMazivila, SarmentoBijak, MichałMazurkiewicz, ŁukaszBikov, AndrásMcGinniss, JohnBilic-Ćurčić, InesMcLean, MaryBilski, JanMcMahon, GerardBingle, LynneMeana, ClaraBiondi, GiuseppinaMeans, Robert T.Bisen, Prakash S.Medina-Puche, LauraBisti, SilviaMehariya, SanjeetBlackburn, GavinMelcangi, Roberto CosimoBlasco, JosefinaMendez, Kevin M.Blazewicz, AnnaMeraviglia, SerenaBlue, Gillian M.Merighi, StefaniaBlume, CorneliaMerlotti, DanielaBoard, MaryMessier, ClaudeBoarescu, Paul-MihaiMessina, Maria AnnaBocca, CinziaMessore, AntonellaBochenek, MatthewMethling, KarenBocianowski, JanMetzinger, LaurentBogdanov, PatriciaMevers, EmilyBonardi, AlessandroMevy, Jean-PhilippeBoros, LaszloMeyer Zu Heringdorf, DagmarBorovac, Josip A.Michalczyk, MałgorzataBorycka-Kiciak, KatarzynaMichels, Alexander J.Bosco, GerardoMiękus, NataliaBouitbir, JamalMigas, LukaszBouret, SebastienMihali, CristinaBove, CeciliaMijić, PeroBoyle, Kristen E.Mikaelyan, Arsen S.Braeckman, BartMilanski, MarcianeBravo, Susana B.Mill, Jose GeraldoBrčić Karačonji, IrenaMiller-Kasprzak, EwaBreves, GerhardMills, KenBroadbent, James A.Minetti, GiampaoloBrodmann, MarianneMiotto, PaulaBroeckling, CoreyMirabelli, MariaBrooks, George A.Mishkovsky, MorBroughton, RichardMishra, DushyantBruzzese, DarioMitani, ShoheiBryniarski, KrzysztofMitulovic, GoranBuck, LeslieMiyazato, HironariBudani, Maria CristinaMlynarz, PiotrBudić-Leto, IrenaMo, ChenglinBuechler, ChristaMoisa, CristianBueschl, ChristophMok, Daniel Kam-WahBufo, Sabino AurelioMollica, AdrianoBulet, PhilippeMontagner, MarcoBusto, RebecaMontecucco, FabrizioButler, MichaelMontero, OlimpioBuzzetti, ElenaMonti, MarcoByrne, HughMookherjee, AbhigyaCaba, OctavioMoon, Jong-SeokCacciola, FrancescoMorales, Jose ManuelCafasso, DonataMorales-Suárez-Varela, María M.Cai, KaiMoral-García, José EnriqueČakić Semenčić, MojcaMoreira, Ana CarolinaCalabrò, PaoloMorgan, JohnCalado, Cecília R. C.Morikawa, ShuntaroCalcaterra, AndreaMorini, LucaCalderón De La Barca, Ana MaríaMoruzzi, NoahCallejón Leblic, BelénMoser, OthmarCalvano, Cosima DamianaMoškon, MihaCampanella, AlessandroMotohashi, KenCancello, RaffaellaMotta, AndreaCano, AinaraMouille, GregoryCanonico, FrancescoMozos, IoanaCapellades, JordiMpofu, EliasCappai, Maria GraziaMucalo, AnaCaretto, SofiaMuhle-Goll, ClaudiaCarlson, RossMukherjee, RukminiCarotti, SimoneMulet, Jose M.Carpentieri, AndreaMüller, GünterCarr, Carolyn A.Muller, MajonCarrer, AlessandroMuller, SebastianCarrera Fernández, CeferinoMurphy, RonanCasal, SusanaMurru, ElisabettaCasas, FrançoisMurthy, DivyaCasassa, Luis FedericoMurzina, Svetlana A.Cascella, RaffaellaMusiał, KingaCásedas, GuillermoMutinelli, FrancoCastellani, ChristophMycielska, MariaCastrucci, Ana MariaNadai, ChiaraCavallari, EleonoraNadler, Jerry L.Cavill, RachelNagai, KeisukeCayre, MyriamNagai, NorihiroCazzola, RobertaNagy, TiborCedars, AriNajdekr, LukášCerrato, AndreaNakazawa, MitsuruČesonienė, LaimaNara, AtsukiCha, SangwonNascimiento, Luana Beatriz Dos SantosChakraborty, ArupNash, SarahChakraborty, SarojNasteska, DanielaChaleckis, RomanasNaylor, KariChan, JoshuaNealon, Nora JeanChan, StanleyNebija, DashnorChang, Jia-FengNedyalkova, MiroslavaChang, Yu-ChaoNeedham, Lisa-MariaChao, Hsu-WenNeffe, SlawomirCharco, Jorge M. CharcoNesterkina, MariiaChatterjee, Som S.Nestor, CaseyChattoraj, ShyamtanuNeumann, ElizabethChaurand, PierreNeunert, GrazynaChauvie, StephaneNeut, ChristelChaveroux, CedricNiculau, Edenilson Dos SantosChavez-Santoscoy, Rocio AlejandraNiculescu, LoredanChekmeneva, ElenaNiemcunowicz-Janica, AnnaChen, Pao-HuanNikinmaa, MikkoChen, Po-HanNikitas, NikitasChen, Shuen-EiNikolaev, EugeneChen, XiaozhuoNikolova, GalinaCheng, Chia-WeiNirala, Niraj K.Cheng, Hong ShengNisticò, Steven PaulCheng, Juei-TangNoce, AnnalisaCheng, Yuan-BinNomura, MasatoshiCherix, AntoineNotas, GeorgeChetverikov, SergeyNovak Kujundžić, RenataChiang, Meng-TsanNowak, DorotaChiavaroli, AnnalisaNtaikou, IoannaChicco, AdamNtana, FaniChiesa, MattiaNtatsi, GeorgiaChing, JianhongNunes, AlexandraChiu, Ching-FengNunez, OscarChiu, Ken C.Nusca, AnnunziataCho, ClaraObeso, DavidCho, Joo-YounOchota, MałgorzataCho, Man-HoOczkowski, MichałCho, YunhiOgihara, Mari H.Choi, KihwanOgrodowczyk, AnnaChong, Suet YenOh, JieunChrist, BastienOh, Sang-IkChristoforides, EliasOhnishi, MasatoshiChrodia, Mahendra D.Okahashi, NobuyukiChrzanowski, ŁukaszOlaya Abril, AlfonsoChuang, Kuo-PinOlchowik-Grabarek, EwaChung, Hee-ChunOlech, MartaChyau, Charng-CherngOlennikov, DaniilChyuan, I-TsuOliveira, Maria ConceiçãoCianci, PasqualeOliveira, Regina VincenziCianciulli, AntoniaOlkowski, BoguslawCiani, FrancescaOnida, SarahCicco, SebastianoOpatovsky, ItaiCicero, ArrigoOpletal, LubomirCincović, Marko R.Ordak, MichalCipakova, IngridOrta, Manuel LuísCiura, KrzesimirOrtiz-Alvarez, LourdesClements, PeterOse, JenniferCocucci, MaurizioOsredkar, JoškoCodella, RobertoOsuna, EduardoCoe, Christopher L.Otelea, MarinaComan, CristinaOthman, AlaaConstantinescu, CaiusOtto, AngelaCorbet, CyrilOuled-Haddou, HakimCorletto, FedericoOzbalci, CagakanCornu, AgnesOziembłowski, MaciejCorrea-Rodríguez, MariaPaar, MargretCortis, CristinaPadkina, Marina V.Costanza, BrunellaPaeng, KijungCostanzo, MichelePaggetti, JeromeCouderc, FrançoisPaglia, GiuseppeCourant, FrédériquePagliarini, David J.Covington, JamesPaldánius, Päivi MariaCox, JanePalermo, AmeliaCozzolino, DanielPalladini, AlessandraCrispo, FabianaPalma, AlessandraCroce, Anna CletaPalmerini, MariaCrofts, CatherinePanatala, RadhakrishnanCrook, NathanPanczyk, TomaszCruz-Sosa, FranciscoPannkuk, EvanCumeras, RaquelPapadopoulos, GeorgiosCunningham, Emma L.Papageorgiou, Ismini E.Curtis, MarionPapassotiriou, Ioannis G.Cuypers, BartPapic, NevenCzumaj, AleksandraPapiris, Spyros A.Czyż, JarosławPappan, KirkCzyż, KatarzynaParada-Turska, JolantaD′Alessandro, AngeloParamio, Jesus M.D′Aloja, ErnestoParamythiotis, SpirosDadáková, KateřinaPareek, ChandraDahiya, RajivPark, IlwooDaly, RónánPark, Jae-HyungDama, MuraliPark, Ji WanDamaziak, KrzysztofPark, JongsunDamiano, FabrizioPark, JulienDaniele, AuroraPark, Jun HyoungDanilowicz-Szymanowicz, LudmilaPark, SangkyuDarvishi, FarshadPark, WooyeongDas, NupurParonetto, Maria PaolaDe Alba, Ángel Emilio MartínezParsons, Peter G.De Araujo, Heloisa Sobreiro SelistreParus, AnnaDe Castro, Raphaela GeorgParvin, KhurshedaDe Felice, ElenaPașca, Aurelian-SorinDe Graaf, Albert A.Passera, AlessandroDe Joussineau, CyrillePateiro, MirianDe Oliveira, Camila MacielPatel, Kashyap A.De Sá Perestrelo, Rosa MariaPatoulias, DimitriosDe Santis, SerenaPatra, AmiyaDe Sotto, RyanPaul, SudipDe Tullio, PascalPavlović, HrvojeDeb, SubrataPawełczyk, SławomiraDeda, OlgaPawlak, KrystynaDei Cas, MichelePawlas, NataliaDeidda, MartinoPechova, AlenaDekker, MarloesPedraza-Chaverri, JoséDel Boccio, PieroPellegrini, ManuelaDel Val, Ioscani JimenezPelosi, LudovicDelgado-Calle, JesusPelucchi, SaraDella Pepa, GiuseppePeng, FoenDelsante, MarcoPenna, FabioDemarquoy, JeanPenno, MeganDeme, PragneyaPenson, Peter E.Denzel, Martin S.Perdomo, GermanDepalma, Ralph G.Pérez, SalvadorDernovics, MihalyPérez-Gálvez, AntonioDeryusheva, SvetlanaPérez-Jiménez, JaraDettmer-Wilde, KatjaPerkovic, Matea NikolacDeutsch, AlexanderPeron, GregorioDevkota, Hari PrasadPerrone, GiancarloDewapriya, PradeepPerrone, Marco AlfonsoDewulf, Joseph P.Persaud, KrishnaDey, AdwitiaPetca, RǎzvanDi Cocco, Maria EnricaPéter, MukliDi Filippo, LuigiPeters, KristianDi Filippo, MarziaPetersmann, AstridDi Gioia, MarcoPeterson, GregoryDias, Fernanda F. G.Pétriacq, PierreDias, FranciscaPetrotos, KonstantinosDias, OscarPetrova, BoryanaDidangelos, TriantafyllosPetruczynik, AnnaDiehl, LindaPhalak, PoonamDíez-López, IgnacioPhan, Thi Tuong VyDimitriadis, GeorgePiestansky, JurajDing, KeyuePillai, NishithaDing, MingPiñeros, AnnieDirckx, NaomiPinto, Diana CláudiaDitonno, PasqualePinto, JoanaDittmann, ElkePinto, LuísDjokovic, RadojicaPiras, CristinaDobrowolny, GabriellaPiri-Moghadam, HamedDocimo, LudovicoPitchika, VinayDokudovskaya, SvetlanaPitti, Julio AlvarezDolenšek, JurijPlacek, KatarzynaDominguez Vías, GermanPłatek, Anna E.Dominguez, RubenPlewa, SzymonDong, FangcongPłotka-Wasylka, JustynaDong, MeixuePlotnikov, EgorDos Santos, José Cleiton SousaPoeggeler, Burkhard H.Dos Santos, Klinsmann CaroloPolesel, JerryDotsikas, YannisPolettini, AlessandraDovnik, AndrazPolis, BaruhDragonieri, SilvanoPomastowski, Paweł P.Drasar, Pavel B.Ponnu, JathishDrougard, AnnePonzini, ErikaDryahina, KseniyaPopescu, Costin-IoanDu, QianPorto, BeatrizDubey, AbhinavPosada-Ayala, MaríaDubrac, SandrinePotaczek, Daniel P.Duda, Marcel MateiPotęga, AgnieszkaDufresne, MartinPotes, YaizaDührkop, KaiPott, Delphine M.Dunić, Jasenka AntunovićPottoo, Faheem HyderDunisławska, AleksandraPoulin, RemingtonDuque, PatriciaPourafshar, ShirinDutta, SumanPozzato, NicolaDvorakova, Magdalena ChottovaPrabhakar, AditiDzidic, AlenPrado, SamiraEbensperger, GermánPrag, Hiran A.Ebert, BirgittaPratesi, AlessandroEchauri, Sergio GarciaPreiner, DarkoEghbalnia, Hamid R.Prieto, MiguelEid, NabilPrince, ElliottEisenstein, EdwardPriyadarshini, MedhaEl Bounkari, OmarPrnová, Marta Š.Elliott, BradleyProbert, FayEllis, Jessica M.Prudent, MarionElnaggar, MehrezPu, XiaopingElolimy, Ahmed A.Puchalska, PatrycjaEnjalbert, FrancisPunia, SnehEnko, DietmarPuślecki, MateuszEras, JordiPyzik, MichalErjavec, IgorQuadri, Syed A.Ernst, MadeleineQuek, Lake-EeEscolà-Gil, Joan CarlesQuéro, AnthonyEscribese, María M.Quintás, GuillermoEspinosa-Escalona, Berta AlejandraRacay, PeterEspitalier, FabienRachwal, KamilaEs-Safi, Nour EddineRadzki, RadosławEssid, Sumia MohamedRafacho, AlexEstevez, AnaRago, DanielaEsumi, TomoyaRaheb, ShariEvangelisti, EdouardRahman, Anas M. AbdelFanizzi, Francesco PaoloRajarapu, Swapna PriyaFarago, PaulRamalho-Santos, JoãoFatima, TahiraRamalingam, NaveenFattuoni, ClaudiaRamchandani, DivyaFaustini, MassimoRamírez, Manuel SánchezFauvelle, FlorenceRamirez-Fernandez, M. d. M.Fazzari, MarcoRammos, ChristosFedrigo, MarnyRamona Moise, AdelaFekete, AgnesRamos-Molina, BrunoFell, DavidRampanelli, ElenaFeng, Chao-HuiRasmusson, Allan G.Feng, Chia-HsienRaspanti, MarioFeofanova, E. V.Rathbone, Alex J.Fernández, IgnacioRathore, Atul SinghFernández-Galilea, MartaRatiu, AndreeaFerreira, António Eduardo N.Rébufa, CatherineFerreira, Antônio GilbertoRege, JuileeFerreira, Carlos Miguel HenriquesReggiani, FrancescaFerreira, NelsonReher, RaphaelFerreira, RodrigoReiff, TobiasFerreiro-González, MartaReis, Cristiano E. RodriguesFiamoncini, JarleiReis, Fernando M.Filice, MariacristinaReis, Ricardo AndradeFilipek, AgnieszkaReiss, Allison B.Fingerhut, RalphRemšík, JánFionda, BrunoRenu, SankarFlament, JulienReunanen, JustusFlibotte, JohnRevelsky, AlexanderFlora, GaganRibo, SilviaFogacci, FedericaRiboni, NicolòForoutan, AidinRichter, SusanForsberg, MatthewRiera, MartaFoster, Derek M.Rijavec, MatijaFranco, PierfrancescoRinschen, MarkusFranzoni, GiuliaRios Solis, LeonardoFriedecky, DavidRizzo, CarmenFriščić, MajaRobak, MałgorzataFukuba, TatsuhiroRobitzsch, AlexanderFukumoto, YoshihiroRoca, MartaFukushima, AtsushiRocchetti, GabrieleFunk, Ryan S.Rocchiccioli, SilviaGać, PawełRocha, MiguelGaigneaux, AnthoulaRochfort, KeithGajger, Ivana TlakRodrigues, JoanaGalal, Azza A. A.Rodrigues, MárcioGalego, LudovinaRodrigues, SophieGallage, SuchiraRodríguez, JaimeGallardo, EugéniaRodriquez, ManuelaGallo, MonicaRogachev, ArtemGambin, AnnaRogina, BlankaGan, PeihengRojo Tirado, Miguel ÁngelGanesan, A.Rojo, Maria AngelesGao, QinRolla, GiovanniGarattini, EnricoRom, OrenGarcia-Garcia, IsidoroRomero, AlejandroGarcía-Rodríguez, AserRosa, Juliana M.Gardón, Juan CarlosRosi, AntonellaGarg, NehaRossi, FrancaGarner, CharlesRossmeislová, LenkaGarten, AntjeRostoker, GuyGaßler, NikolausRoubertoux, PierreGaude, EdoardoRouger, CarolineGaul, DavidRoverso, MarcoGaur, PankajRoy, Swapan KumarGaviglio, AmyRozalski, MarcinGaworski, MarekRuoppolo, MargheritaGe, XiaodongRupnik, Marjan SlakGelb, MichaelRuscica, MassimilianoGeorgiana, MartisRussell, Steve T.Georgiou, IoannisRusso, IsabellaGerm, MatejaRuta, LaviniaGervasoni, JacopoRuzza, PaoloGevi, FedericaRychahou, Piotr G.Ghadieh, Hilda E.Rydosz, ArturGhassemi Nejad, JalilSaddala, Madhu SudhanaGheibi, SevdaSaha, Sushanta KumarGhesquiere, BartSaito, KosukeGhosh, ChiranjitSakai, ShotaGhosh, DebashisSakamoto, AtsushiGhosh, SharmilaSakurada, YoichiGhosh, SoumitaSalamon, DominikaGiacomelli, ChiaraSalehzadeh-Yazdi, AliGiangregorio, NicolaSalek, RezaGiebultowicz, JoannaSalles, ChristianGika, HelenSalman, MootazGil, Andrea Paola RojasSalomone, AlbertoGiller, KatrinSalto-Gonzalez, RafaelGil-Solsona, RubénSalvo, AndreaGines Ruiz, RafaelŠamec, DunjaGiromini, CarlottaSamiec, MarcinGiron-Gonzalez, Maria D.Sampaio, Bruno LeiteGiunchi, FrancescaSampaio-Marques, BelémGiussani, PaolaSamsonov, AlexeyGlibowski, PawełSánchez Guijo, AlbertoGlobisch, DanielSánchez López, ElenaGoddard, Mary-LorèneSanchez-Alcazar, JoseGombarska, DanielaSandhu, AmandeepGomez Roldan, Maria VictoriaSandín-España, PilarGómez, CristinaSándor, BarbaraGoncalves, Luís GafeiraSango, KazunoriGontier, EricSantacroce, LuigiGonzalez, ManuelSapina, MatejGonzalez-Bulnes, AntonioSaravanakumar, KandasamyGonzález-De-Peredo, Ana V.Sarkar, DepanjanGonzález-Domínguez, RaúlSarode, Gaurav V.Goodson, MichaelSase, AjinkyaGorbacheva, LiubovSateriale, AdamGordeeva, OlgaSato, ShuzoGore, Andrea C.Saunders, DianeGoričar, KatjaSaura-Sanmartin, AdrianGornicki, KrzysztofSaurav, KumarGoroncy, Alexander K.Savu, AnamariaGorynski, KrzysztofSayed, Ibrahim M.Goudarzi, MaryamScacco, SalvatoreGovus, AndrewSchipke, Jochen D.Graham, StewartSchlein, ChristianGrandinetti, AndrewSchlüter, Klaus-DieterGranica, SebastianSchmidt, Cameron A.Grant, DavidSchmoll, DieterGreer, DennisSchrama, DeniseGregorini, MarilenaSchripsema, JanGrelet, SimonSchubert, RudolfGrieco, FrancescoSchuhmacher, RainerGrilli, AlfredoSchweizer, MichaelGrimaldi, PaolaŚciskalska, MilenaGrinzato, AlessandroSciubba, FabioGrist, JamesScott, Fraser W.Gromadzka, GrażynaScudiero, RosariaGrösch, SabineSeifert, StephanGross, Jane E.Selvakesavan, RajendranGrumati, PaoloSengupta, ArjunGrzesiak, MalgorzataSeow, Kok-MinGrzybowska, EwaSepulveda, NéstorGu, ZhenglinSerefko, AnnaGuagnano, Maria TeresaSerkan Selli, SerkanGuerini, Franca R.Serra, RaffaeleGuevara, NataliaSertić, MirandaGuha, SujaySessa, FrancescoGuirimand, GregorySeto, Sai-WangGunaje, JayaramaSeverino, PaoloGurzov, EstebanShaposhnikov, MikhailGutierrez-Juarez, RogerSharma, DivyaGwak, Ho-ShinSharma, GauravHabili, NuredinSharma, RavindraHaga, SatoshiSheikh, BilalHageman, JosShelley, MackHai, QiminShen, Hongying (Hoy)Hajime, HiraseSher, FarooqHalachmi, SarelSherman, ArthurHalama, AnnaShi, Han-pingHamano, HirofumiShibaeva, Tatjana G.Hamid, ZeeshanShiraishi, Jun-ichiHan, DongShizu, RyotaHan, HongweiShukla, KirtikarHandakas, EvangelosShulman, Lee P.Hannani, DalilSiamwala, Jamila H.Hannibal, LucianaSidorov, Evgeny V.Hano, TakeshiSikalidis, AngelosHansen, Esben Søvsø SzocskaSilva, AndreHao, FuhuaSilva, AuroraHardt, MarkusSilva, CatarinaHarms, AmySilva, Margarida F. B.Harshfield, Eric L.Silva, Saulo LuzHarvey, Elizabeth L.Silvagno, FrancescaHasunuma, TomohisaSimitzis, PanagiotisHayes, AlanSimova, SvetlanaHe, LixiaSingh, Amit K.Hebbard, LionelSingh, Rupesh KumarHeberger, KarolySipeki, NóraHedemann, Mette SkouSipos, KatalinHellingwerf, Klaas J.Sisti, DavideHemachandra, Reddy P.Sivanesan, IyyakkannuHemeryck, LieselotSkeffington, Katie L.Hendrickx, Diana M.Skelin Klemen, MašaHennebelle, MarieSkonieczna-Żydecka, KarolinaHermesch, SusanneSkowrońska, MonikaHermida-Prieto, ManuelSkrzypski, MarekHernández-Núñez, EmanuelSleno, LekhaHesami, MohsenSlijkhuis, NuriaHeux, StéphanieSmilde, Age K.Hilhorst, MarcSmith, KerryHindié, ElifSnider, Ashley J.Hinkley, James MatthewSnowden, Stuart G.Hirasaka, KatsuyaSoba, DavidHirayama, AkiyoshiSobolewski, CyrilHofer, JohannesSobotka, LubosHogenEsch, HarmSokol, MariaHolcapek, MichalSolano, FranciscoHomann, ThomasSoldan, Samantha S.Honneffer, Julia B.Solimando, Antonio G.Honrao, ChandrashekharSolís Sanchez, GonzaloHorhat, DeliaSołowski, GawełHori, MikaSomlyai, GáborHorn, PatrickŠoša, IvanHorvat, DanielaSoto, Esther CuadradoHossain, EkramSousa, Sílvia A.Hossain, MotaherSpakowicz, Daniel J.Hoste, LeviSpégel, PeterHotea, IonelaSreedharan, SreejeshHotos, George N.Stacchiotti, AlessandraHou, Chih YaoStåhlman, MarcusHoughton, Lauren C.Stančiaková, LuciaHoussier, MarianneStanimirova, IvanaHrubá, PetraStanstrup, JanHsu, Chin-LinStarkova, JuliaHsu, Jih-TayStavrianidi, AndreyHu, BinStawny, MaciejHuang, Alex L.Steenblock, CharlotteHuang, ChaoqunStefaniuk, DawidHuang, Chien-JuiStefanowicz, PiotrHuang, Chung-HsiungStepien, Karolina M.Huber, GaspardSteubl, DominikHuber, ReneSteuer, Andrea E.Hulina-Tomašković, AndreaStevens, RickHurley, James B.Steyn, Adrie J. C.Huynh, KevinStiburkova, BlankaHwang, Eun SeongStietz, Kimberly P. KeilHwang, Jung ShanStobiecki, MaciejHyšpler, RadomírStocchero, MatteoIaccarino, NunziaStock, Naomi L.Iacoviello, MassimoStompór, TomaszIdol, TravisStraub, LeonIentile, RiccardoStrauß, MarkusIglesias-Osma, Maria CarmenStres, BlažIkonomopoulou, MariaSubra, Jean-FrançoisImperatore, RobertaSubramanian, ChitraIndini, AliceSugiki, ToshihikoInfascelli, FedericoSukumaran, VijayakumarInoue, DaisukeSumin, Alexei N.Inyushin, Mikhail Y.Summers, Katie LynnIorio, EgidioSun, LipingIppolito, AntonioSunayama, HirobumiIrfan, MuhammadSuzuki, KeiichiroIshibashi, ShunSwarup, SanjayIshikawa, ManabuSydor, SvenjaIvanovska, NinaSynowiec, AgnieszkaIzawa, Kazuhiro P.Szablewski, LeszekJablan, JasnaSzafranek-Nakonieczna, AnnaJackson, Joshua G.Széliová, DianaJacobo-Velazquez, DanielSzeremeta, MichalJacome-Sosa, MiriamSzutowicz, AndrzejJágr, MichalSzyszkowicz, MieczyslawJakobek, LidijaTabernero-Duque, María-JesúsJakovcevski, IgorTaiar, RedhaJamka, MałgorzataTakahashi, MasamichiJang, CholsoonTakis, Panteleimon G.Jang, SeongjaeTan, Bee K.Janikowska, GrażynaTan, LiJanker, LukasTănase, Ana-MariaJankevics, AndrisTang, Peter H.Jans, Judith J. M.Tankiewicz-Kwedlo, AnnaJanuskaitiene, IrenaTata, AlessandraJaumot, JoaquimTava, AldoJenkins, BenjaminTea, IllaJeong, JaesikTebani, AbdellahJerzy, MosiewiczTeixeira, Miguel CachoJeucken, AikeTelatin, AndreaJewell, Dennis E.Tenore, GianlucaJia, BaoleiTeodoro, JoãoJiang, Shu-YeTeragawa, HirokiJimenez-Chillaron, JosepTetz, GeorgeJimenez-Palomares, MargaritaThackray, AlanaJolicoeur, MarioThomasset, BrigitteJones, JaceThomeer, MichielJordan, Bénédicte F.Thongprayoon, CharatJoshi, ChintanTilea, IoanJoubert, AnnieTimpani, CaraJuan, Yung-ShunTodisco, SimonaJung, FriedrichTomaszewska, EwaJurić, SlavenTonazzi, AnnamariaJurkonienė, SigitaTormo, RubenKaether, ChristophTorras, JuanKahlert, StefanTorras-Ambròs, JoanKalemba-Drożdż, MałgorzataTorta, FedericoKalíková, KvětaTotta, PierangelaKallithraka, StamatinaTrcek, JanjaKalogirou, Andreas S.Trefely, SophieKanagendran, ArooranTrifan, AdrianaKanaya, MoekoTrimigno, AlessiaKaneko, GenTripathi, AnupriyaKanellos, PanagiotisTripisciano, CarlaKaňková, KateřinaTrognitz, FriederikeKapritsou, MariaTroncoso-Ponce, AdrianKarasawa, KenTrucco, MassimoKardassis, DimitrisTsingotjidou, AnastasiaKarimian-Jazi, KianushTun, Mya Myat NgweKarolyi, DanijelTurner, JustineKashihara, NaokiTuttolomondo, AntoninoKasprzak, AldonaUchimura, KoheiKasprzak, KamilaUlmer, Candice Z.Kaszaki, JozsefUlrich, BenjaminKatalinić, MajaUrsache, RobertasKatayama, SatoshiVafiadaki, ElizabethKatsafadou, AngelikiVaismoradi, MojtabaKatselis, GeorgeValenzuela, StellaKaushik, PrashantValles, Soraya L.Kawabata, ShinjiValletta, AlessioKawamukai, MakotoVamanu, EmanuelKay, RichardVamvakaris, IoannisKelleher, Joanne K.Van Dam, NicoleKepinska, MartaVan Grevenynghe, JulienKeshari, Kayvan R.Van Gurp, LeonKessal, KarimaVan Weeghel, MichelKeswani, SundeepVaradarajan, SudhaharKhallouki, FaridVarì, Maria RosariaKhan, IrfanVarillas Delgado, DavidKhan, Md. Rajibur RahamanVarvarigou, AnastasiaKhan, Mohammad ImranVavitsas, KonstantinosKhan, MohsinVeilleux, AlainKhan, RayyanVelan, SendhilKhemtong, ChalermchaiVelena, AstridaKholová, IvanaVelickovic, DusanKicel, AgnieszkaVella, Filomena MonicaKiefer, PatrickVergara, DanieleKieliszek, MarekVermathen, MartinaKikionis, StefanosVetere, AmedeoKikuchi, HaruhisaVidkjær, Nanna HjortKilk, KalleVigors, StaffordKim, Dong-HyunVinayaka, Ajjampura C.Kim, HoonVincent, John BertramKim, Hyeong-GeugVirgen-Ortíz, Jose J.Kim, Jae KwangVirto, MailoKim, JoonhoonVon Eggeling, FerdinandKim, Ki-TaeVrachatis, Dimitrios A.Kirienko, NatashaVuorio, AlpoKirwan, JenniferWagmann, LeaKite, Geoffrey C.Wakiguchi, HiroyukiKlavins, KristapsWanders, RonaldKleeff, JörgWang, Chih-HaoKleindienst, AndreaWang, Feng-ShengKleszcz, RobertWang, GuanKnupp, GerdWang, HaoKo, Frank C.Wang, HsiuyingKobayashi, SatoruWang, JiongweiKodani, EitaroWang, Kai-LeeKoeffel, ReneWang, LeiKoelmel, JeremyWang, Mong-HengKöfeler, Harald C.Wang, Shu-PingKoh, Cho YeowWarzych-Plejer, EwelinaKoike, ShinWasniewska, MalgorzataKoizume, ShiroWatanabe, EiichiKokokiris, LambrosWatson, DavidKokoszyński, DariuszWeinhold, AlexanderKolwicz, Stephen C.Whitfield, PhilKomives, TamasWidemann, EmilieKonnikova, YelizavetaWidrlechner, MarkKonno, HiroyukiWilde, Michael J.Kontoudakis, NikolaosWilson, Alphus D.Korach-André, MarionWilson, Philippe B.Kornilov, SergeyWiszniewska, AlinaKosacka, JoannaWójciak, MagdalenaKoshinaka, KeiichiWojciechowska-Solis, JuliaKosicka-Noworzyń, KatarzynaWojtunik-Kulesza, Karolina A.Kosma, Ioanna S.Wojtyla, ŁukaszKostov, GeorgiWong, AlanKot, Anna MariaWong, Man-SauKotula-Balak, MałgorzataWood, Paul L.Koudounas, KonstantinosWoods, Jon P.Kovinich, NikWorthmann, AnnaKowalski, RadosławWu, ChaoKozłowska, MariolaWu, Ping-HsunKoźmińska, AleksandraWu, Shi-BeiKrajewska-Włodarczyk, MagdalenaWu, Wen-TzuKramer, HolgerWysocka-Mincewicz, MartaKrishna, SmritiXie, LingxiaoKról, EwelinaXu, DeyangKrssak, MartinYadav, DhananjayKrüger, MarcusYamamoto, MasayukiKu, Kang-MoYamashita, AtsushiKubo, AtsushiYanda, MuraliKučera, LukášYang, Chao-YieKüken, AnikaYang, HongshunKuliczkowski, KazimierzYang, KunKulik, AnnaYao, Pei-LiKultima, KimYe, MingyuKumar, Amit (Canada)Yegles, MichelKumar, Amit (USA)Yilmaz, AliKupczyński, RobertYoon, DahyeKurhaluk, NataliaYou, Hye JinKuryłowicz, AlinaYoung, AlixKusunoki, KazutakaYousefi, Behrooz HooshyarKusztal, MariuszYu, Dan-XiaKwiecien, IngaYu, KitaokaLa Frano, MichaelYung, Hong-WaLaaksonen, ReijoYusa, KosukeLabanca, FabianaZacchia, MiriamLabudda, MateuszZacharias, Helena U.Laganà, Antonio SimoneZagrebelsky, MartaŁagowska, KarolinaZając, DominikaLagrange, JeremyZakrocka, IzabelaLakatošová, SilviaZanella, IsabellaLake, LachlanZarei, ImanLalia, AntigoniZarrine-Afsar, ArashLalk, MichaelZawadzka, MalgorzataLalowski, MaciejZbroch, EdytaLamichhane, SantoshŽdralević, MašaLança, Maria JoãoZeljkovic, SanjaLancaster, Graeme I.Zemła, JoannaLandi, SilviaZeng, HuaweiLange, EwaZhang, JialingLanger, HenningZhao, JingyiLanning, NathanZhao, LiangLapucci, AndreaZhu, LinLarrouy-Maumus, GeraldZhukov, IgorLasonder, EdwinZhuo, ChunliuLau, ChunghoZieminska, ElzbietaLau, Chung-Ho E.Zieseniss, AnkeLauberts, MarisZipperle, JohannesLauterbach, RyszardZucchi, RiccardoLaw, Henry Chun HinZurita, Mario

